# VISUALIZING AGING EQUITY: CROSS-SECTORAL PARTNERSHIPS TO CREATE MAPPING TOOLS FOR SOCIAL CHANGE

**DOI:** 10.1093/geroni/igad104.0708

**Published:** 2023-12-21

**Authors:** Clara Scher, Emily Greenfield, Rodney Harrell

## Abstract

This symposium will feature ways in which gerontologists are advancing the development of online, interactive mapping tools on aging. Utilizing GIS mapping technology and leveraging cross-sectoral partnerships, the speakers will present case illustrations of efforts to develop aging-focused mapping tools that aggregate data sources at the municipal, state, and national levels to advance a spatial awareness of supports for older adults across diverse communities. First, as an example from the nonprofit sector, the New York Academy of Medicine will present on IMAGE:NYC, The Interactive Map of Aging. This presentation will describe the utility of this tool for policymakers, planners, researchers, and social service providers. Representing work from within the academic sector, the Rutgers School of Social Work will describe an academic-public sector partnership to adapt the IMAGE:NYC map for two counties in New Jersey. Next, presenters from the University of North Texas will describe their partnership with the City of San Antonio to develop an intelligent demand responsive transportation system for older adults and individuals with disabilities. This project involves the use of GIS mapping tools and qualitative research methods to identify place-based transportation gaps and strategies for improvement. Focusing on the national level, the National Council on Aging will describe its work developing mapping tools, including a map that displays area-level rates in eligibility gaps for three federal benefit programs. With an orientation to aging equity, these tools hold promise for fostering inclusive and healthy communities where older adults of diverse intersectional socio-spatial positions can age well. This is an Environmental Gerontology Interest Group Sponsored Symposium.

